# PI3 kinase directly phosphorylates Akt1/2 at Ser473/474 in the insulin signal transduction pathway

**DOI:** 10.1530/JOE-13-0172

**Published:** 2014-01

**Authors:** A Tsuchiya, T Kanno, T Nishizaki

**Affiliations:** 1 Division of Bioinformation, Department of PhysiologyHyogo College of Medicine1-1 Mukogawa-cho, Nishinomiya, 663-8501Japan

**Keywords:** adipocyte, cell biology, GLUT4, insulin signaling

## Abstract

Insulin stimulated translocation of the glucose transporter GLUT4 from the cytosol to the plasma membrane in a concentration (1 nM–1 μM)-dependent manner and increased glucose uptake in 3T3-L1 adipocytes. Insulin-induced GLUT4 translocation to the cell surface was prevented by the phosphoinositide 3 kinase (PI3K) inhibitor wortmannin, the 3-phosphoinositide-dependent protein kinase 1 (PDK1) inhibitor BX912 or the Akt1/2 inhibitor MK2206, and by knocking-down PI3K, PDK1 or Akt1/2. Insulin increased phosphorylation of Akt1/2 at Thr308/309 and Ser473/474, to activate Akt1/2, in the adipocytes. Insulin-induced phosphorylation of Akt1/2 was suppressed by wortmannin and knocking-down PI3K, while no significant inhibition of the phosphorylation was obtained with BX912 or knocking-down PDK1. In the cell-free Akt assay, PI3K phosphorylated Akt1 both at Thr308 and Ser473 and Akt2 at Ser474 alone. In contrast, PDK1 phosphorylates Akt1 at Thr308 and Akt2 at Thr309. The results of this study indicate that PI3K activates Akt1, independently of PDK1, and Akt2 by cooperating with PDK1 in the insulin signal transduction pathway linked to GLUT4 translocation.

## Introduction

Akt is a serine/threonine protein kinase bearing multiple cellular processes such as glucose metabolism, apoptosis, cell proliferation, transcription, and cell migration. Akt includes three closely related isoforms Akt1, Akt2, and Akt3 (Calera *et al*. 1998). Of the isoforms Akt1 exerts its anti-apoptotic action, thereby promoting cell survival (Song *et al*. 2005). Akt1 is also implicated in protein synthesis, responsible for skeletal muscle hypertrophy and tissue growth (Lai *et al*. 2004). Accumulating evidence has indicated the involvement of Akt1 in many types of cancer (Cheung & Testa 2013). Akt2, on the other hand, plays a pivotal role in glucose homeostasis. Akt2 stimulates translocation of the glucose transporter GLUT4, abundantly expressed in skeletal muscle and fat cells, to the cell surface, causing insulin-induced glucose uptake into cells (Garofalo *et al*. 2003). Akt3 is preferentially expressed in the brain, but its role is not fully understood.

The receptor tyrosine kinase insulin receptor is implicated in the activation of Akt through a pathway along an insulin receptor substrate (IRS)/phosphatidylinositol 3 kinase (PI3K)/3-phosphoinositide-dependent protein kinase 1 (PDK1)/Akt axis (Maarbjerg *et al*. 2011). Insulin activates insulin receptor, to phosphorylate its own receptor and IRS, thereby dissociating IRS from insulin receptor to activate PI3K. The activated PI3K produces phosphatidylinositol (3,4,5)-triphosphate (PI(3,4,5)P_3_) by phosphorylation of phosphatidylinositol 4,5-bisphosphate (PI(4,5)P_2_), and in turn, PI(3,4,5)P_3_ activates PDK1 through its binding. The activation of Akt1/2 is achieved by phosphorylation of Thr308 for Akt1 and Thr309 for Akt2 in the activation-loop of the kinase domain and Ser473 for Akt1 and Ser474 for Akt2 in the carboxy-terminal regulatory region (Yang *et al*. 2002, Song *et al*. 2005). Thr308/309 is phosphorylated by PDK1, which is dephosphorylated by protein phosphatase 2A (PP2A; Vanhaesebroeck & Alessi 2000, Beaulieu *et al*. 2005). The phosphatase and tensin homolog (PTEN) and Src homology 2-containing inositol phosphatase (SHIP) reduce PI(3,4,5)P_3_ by dephosphorylating it into PI(4,5)P_2_ and PI(3,4)P_2_, respectively, to attenuate PDK1 activity followed by Akt1/2 phosphorylation at Thr308/309 (Finlay 2012). The mammalian target of rapamycin complex 2 (mTORC2), that is activated by PI3K (Razmara *et al*. 2013), phosphorylates Akt1/2 at Ser473/474, and the PH domain and leucine-rich repeat protein phosphatase dephosphorylates Akt1/2 at this residue (Bayascas & Alessi 2005, Gao *et al*. 2005). Phosphorylation of Akt1/2 at Ser473/474, alternatively, is achieved by an inhibitor of NFκ-B kinase subunit ε (IKBKE; Guo *et al*. 2011) or P21-activated kinase 1 (Pak1; Mao *et al*. 2008), in a PI3K-independent manner.

This study was conducted to gain further insight into PI3K-induced Akt activation in the insulin signal transduction pathway. To address this, we monitored GLUT4 mobilizations, measured glucose uptake into cells, and assayed Akt1/2 activity in 3T3-L1 adipocytes expressing myc-tagged GLUT4 (3T3-L1-GLUT4myc adipocytes) and under cell-free conditions. We show here that insulin stimulates GLUT4 translocation to the cell surface and increases glucose uptake into 3T3-L1 adipocytes in an Akt1/2-dependent manner; PI3K phosphorylates both Thr308 and Ser473 for Akt1 and Ser474 alone for Akt2; and PDK1 phosphorylates Thr308 for Akt1 and Akt2. This may represent fresh insight into PI3K-regulated Akt1/2 activation in the insulin signal transduction pathway.

## Subjects and methods

### Cell culture

3T3-L1-GLUT4myc fibroblast cell line expressing GLUT4myc is constructed by inserting a human c-MYC epitope (14 amino acids) into the first ectodomain of GLUT4. Cells were cultured in DMEM supplemented with 10% (v/v) calf serum, penicillin (final concentration, 100 U/ml), and streptomycin (final concentration, 0.1 mg/ml), in a humidified atmosphere of 5% CO_2_ and 95% air at 37 °C. When cells had reached confluence (day 0), medium was changed to DMEM supplemented with 10% (v/v) fetal bovine serum (FBS), 1 μM dexamethasone, 0.5 mM 3-isobutyl-metyhl-xanthine, and 0.1 mg/ml insulin to differentiate from fibroblast to adipocytes (3T3-L1-GLUT4myc adipocytes). At day 3, day 7, and day 11, the medium was changed to DMEM supplemented with 10% (v/v) FBS. At day 14, cells were used for experiments. 3T3-L1-GLUT4myc adipocytes were used for all the cell experiments in this study.

### Oil-Red O staining of 3T3-L1 adipocytes

Oil-Red O (Wako Pure Chemical Industries, Osaka, Japan) was dissolved in isopropanol and left over night at room temperature. The solution was diluted with distilled water (6:4) and filtered through a filter paper. Cells on day 0, day 3, and day 14 from differentiation treatment were fixed with 10% (v/v) formalin in PBS for 10 min and stained with the Oil-Red O solution for 20 min at room temperature. Cells were washed with 60% (v/v) isopropanol and then washed twice with PBS. Fat droplets in adipocytes were visualized with a phase contrast microscope.

### Monitoring of GLUT4 mobilizations

3T3-L1-GLUT4myc adipocytes were incubated in Krebs–Ringer-HEPES buffer (136 mM NaCl, 4.7 mM KCl, 1.25 mM CaCl_2_, 1.25 mM MgSO_4_, and 20 mM HEPES, pH 7.5) containing 0.2% (w/v) BSA supplemented with 10 mM glucose for 1 h at 37 °C. Cells were treated with insulin in the presence and absence of inhibitors for 20 min. Then, cells were homogenized by sonication in an ice-cold mitochondrial buffer (210 mM mannitol, 70 mM sucrose, and 1 mM EDTA, 10 mM HEPES, pH 7.5) containing 1% (v/v) protease inhibitor cocktail (Nacalai Tesque, Kyoto, Japan) and subsequently, homogenates were centrifuged at 800 ***g*** for 5 min at 4 °C. The supernatants were centrifuged at 11 000 ***g*** for 15 min at 4 °C and further, the collected supernatants were ultracentrifuged at 100 000 ***g*** for 60 min at 4 °C to separate the cytosolic and plasma membrane fraction. The supernatants and pellets were used as the cytosolic and plasma membrane fractions respectively. Whether the cytosolic and plasma membrane components were successfully separated was confirmed in the western blot analysis using antibodies against the cytosolic marker, lactate dehydrogenase, and the plasma membrane marker, cadherin.

Protein concentrations for each fraction were determined using a BCA protein assay kit (Thermo Fisher Scientific, Waltham, MA, USA). Plasma membrane fraction proteins were resuspended in the mitochondrial buffer containing 1% (w/v) SDS. Proteins for each fraction were separated by SDS–PAGE and transferred to polyvinylidene difluoride (PVDF) membranes. After blocking with TBS-T (150 mM NaCl, 0.1% (v/v) Tween-20, and 20 mM Tris, pH 7.5) containing 5% (w/v) BSA, blotting membranes were reacted with an anti-c-myc antibody (Merck Millipore) followed by an HRP-conjugated goat anti-mouse IgG antibody. Immunoreactivity was detected with an ECL kit (Invitrogen) and visualized using a chemiluminescence detection system (GE Healthcare, Piscataway, NJ, USA). Signal density was measured with ImageQuant software (GE Healthcare).

### Glucose uptake assay

3T3-L1-GLUT4myc adipocytes were incubated in the Krebs–Ringer-HEPES buffer containing 0.2% (w/v) BSA supplemented with 10 mM glucose for 1 h at 37 °C. Then, cells were left untreated or treated with insulin (100 nM) in PBS supplemented with 10 mM glucose for 20–120 min at 37 °C. After treatment, extracellular solution was collected and glucose was labeled with *p*-aminobenzoic ethyl ester (ABEE). Then, 5 μl ABEE-labeled solution was injected onto the column (150×4.6 mm) equipped in the HPLC system. ABEE-labeled glucose was detected at an excitation wavelength of 305 nm and an emission wavelength of 360 nm using a fluorescence detector. The concentration of glucose taken up into cells was calculated by subtracting extracellular glucose concentration after incubation for 20–120 min from initial extracellular glucose concentration (10 mM).

### Construction and transfection of siRNA

The siRNA to silence the Akt1/2-targeted gene (Akt1/2 siRNA) was obtained from Santa Cruz Biotechnology, Inc. The siRNAs to silence the PI3K p85α-targeted gene (PI3K KD), the PDK1-targeted gene (PDK1 KD), and the negative control siRNA (NC) were obtained from Ambion (Carlsbad, CA, USA). The sequences of siRNAs used were 5′-GCGAAUGAUAUGUAUCAGAtt-3′ and 5′-UCUGAUACAUAUCAUUCGCtc-3′ for PI3K p85α, 5′-CCUCGUUUAUGUUUCUGCGtt-3′ and 5′-CGCAGAAACAUAAACGAGGtc-3′ for PDK1. Each NC siRNA had the scrambled sequence, the same GC content, and nucleic acid composition. siRNAs were transfected into the cells using a Lipofectamine reagent (Invitrogen). Cells were used for experiments 48 h after transfection.

### Western blotting

3T3-L1-GLUT4myc adipocytes were incubated in Krebs–Ringer-HEPES buffer containing 0.2% (w/v) BSA, supplemented with 10 mM glucose for 1 h at 37 °C. The cells transfected with and without siRNAs were left untreated or treated with insulin in the presence and absence of inhibitors for 10 min. Next, the cells were lysed in lysis buffer (150 mM NaCl, 20 mM EDTA, 0.5% (v/v) Nonidet P-40, and 50 mM Tris, pH 7.4) containing 1% (v/v) protease inhibitor cocktail and 1% (v/v) phosphatase inhibitor cocktail (Nacalai Tesque), and then centrifuged at 800 ***g*** for 5 min at 4 °C. The supernatant was used as total cell lysate.

For western blotting, proteins were separated by SDS–PAGE and then transferred to PVDF membranes. Blotting membranes were blocked with TBS-T containing 5% (w/v) BSA and subsequently reacted with antibodies against peroxisome proliferator-activated receptor γ (PPARγ) (Cell Signaling Technology, Inc., Danvers, MA, USA), phospho-Thr308/309-Akt1/2 (pT308(9)), phospho-Ser473/474-Akt1/2 (pS473(4)), Akt1/2 (Cell Signaling Technology), Akt1 (Cell Signaling Technology), Akt2 (Cell Signaling Technology), PI3K (Sigma), PDK1 (Sigma), or β-actin (Sigma). After washing, membranes were reacted with an HRP-conjugated goat anti-rabbit IgG or goat anti-mouse IgG antibody. Immunoreactivity was detected with an ECL kit (Invitrogen) and visualized using a chemiluminescence detection system (GE Healthcare). Protein concentrations for each sample were determined with a BCA protein assay kit (Thermo Fisher Scientific).

### Cell-free Akt assay

Human recombinant Akt1 or human recombinant Akt2 (Active Motif, Carlsbad, CA, USA) was reacted without or with His-tagged human recombinant PI3K (p110β/p85α) (Sigma) or His-tagged human recombinant PDK1 (SignalChem, Richmond, BC, Canada), which was purified by affinity chromatography, in the presence and absence of wortmannin or BX912 in a medium containing 25 mM 3-morpholinopropanesulfonic acid (pH 7.2), 25 mM MgCl_2_, 12.5 mM glycerol 2-phosphate, 5 mM ethylene glycol-bis(2-aminoethyl ether)-*N*,*N*,*N*′,*N*′-tetraacetic acid, 2 mM EDTA, 0.25 mM dithiothreitol, and 250 μM ATP at 30 °C for 20 min. Phosphorylated Akt was detected by western blotting using antibodies against pT308(9), pS473(4), and Akt1/2 as described earlier.

### Statistical analysis

Statistical analysis was carried out using unpaired *t*-test, Dunnett's test, and ANOVA followed by a Bonferonni correction.

## Results

### Insulin stimulates GLUT4 translocation to the plasma membrane and increases glucose uptake into cells in an Akt1/2-dependent manner

**Figure 1:**
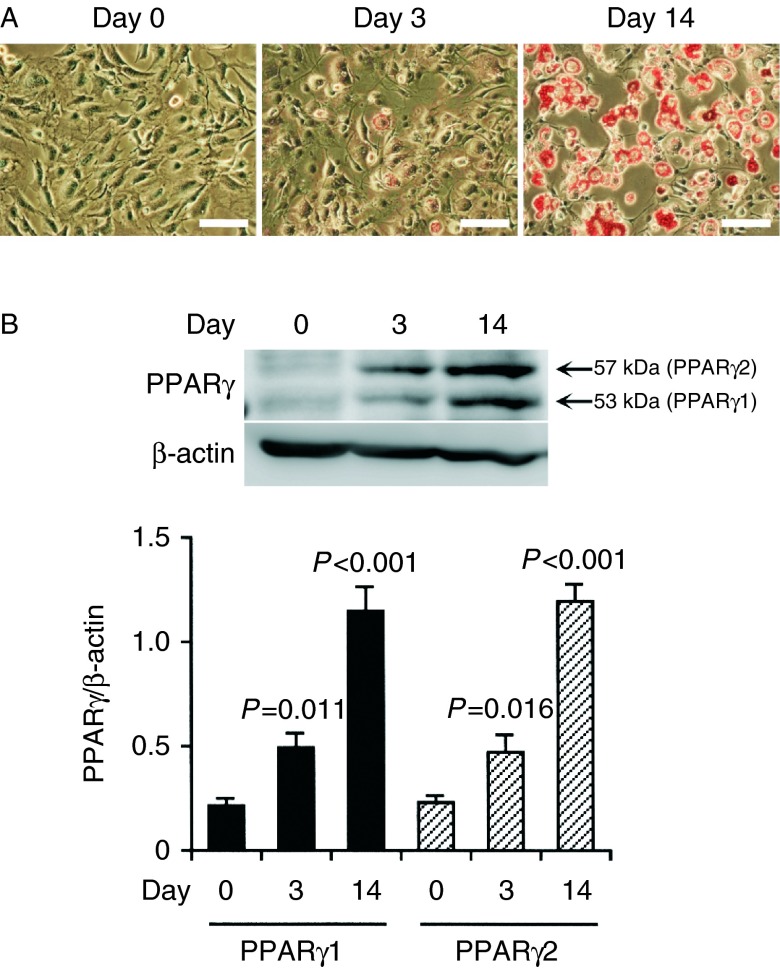
(A) Oil-Red O staining. 3T3-L1-GLUT4myc fibroblasts were stained with Oil-Red O on day 0, day 3, and day 14 after differentiation induction. Bars, 100 μm. Note that adipose droplets in cells are dyed red and that a similar effect was found in four independent experiments. (B) Cells collected on day 0, day 3, and day 14 after differentiation induction were lysed followed by western blotting using antibodies against PPARγ and β-actin. In the blotting membrane shown, an anti-PPARγ antibody produces two signal bands at 53 and 57 kDa, each corresponding to PPARγ1 and PPARγ2. Signal intensities for PPARγ1 and PPARγ2 were normalized to those for β-actin. In the graph, each column represents the mean (±s.e.m.) normalized expression of PPARγ1 and PPARγ2 (*n*=4 independent experiments). *P* values as compared with the expression on day 0, Dunnett's test.

We initially examined whether 3T3-L1-GLUT4myc fibroblasts are differentiated into adipocytes using Oil-Red O. Adipose tissue is stained with Oil-Red O, to detect adipose conversion (Ramírez-Zacarías *et al*. 1992). 3T3-L1-GLUT4myc fibroblasts before differentiation induction (day 0), with spindle-like shapes, had no reaction to Oil-Red O (Fig. 1A). On day 3 after differentiation induction a few cells, which had assumed a round shape, were positive to Oil-Red O and on day 14 almost all the cells, which had assumed a round shape, were dyed red (Fig. 1A). This indicates that 3T3-L1-GLUT4myc fibroblasts are readily differentiated into adipocytes.

To obtain further evidence for adipocyte differentiation, we carried out western blotting using an antibody against PPARγ, an adipocyte-specific marker (Tontonoz *et al*. 1994). Expression of PPARγ1 and PPARγ2 was gradually increased after differentiation induction, and the expression on day 14 after differentiation induction was significantly much higher than that on day 0 (Fig. 1B). This confirms that 3T3-L1-GLUT4myc fibroblasts are actually differentiated into adipocytes on day 14 after differentiation induction. We, therefore, used 3T3-L1-GLUT4myc fibroblasts differentiated for more than 14 days (3T3-L1-GLUT4myc adipocytes) for the ensuing experiments.

**Figure 2:**
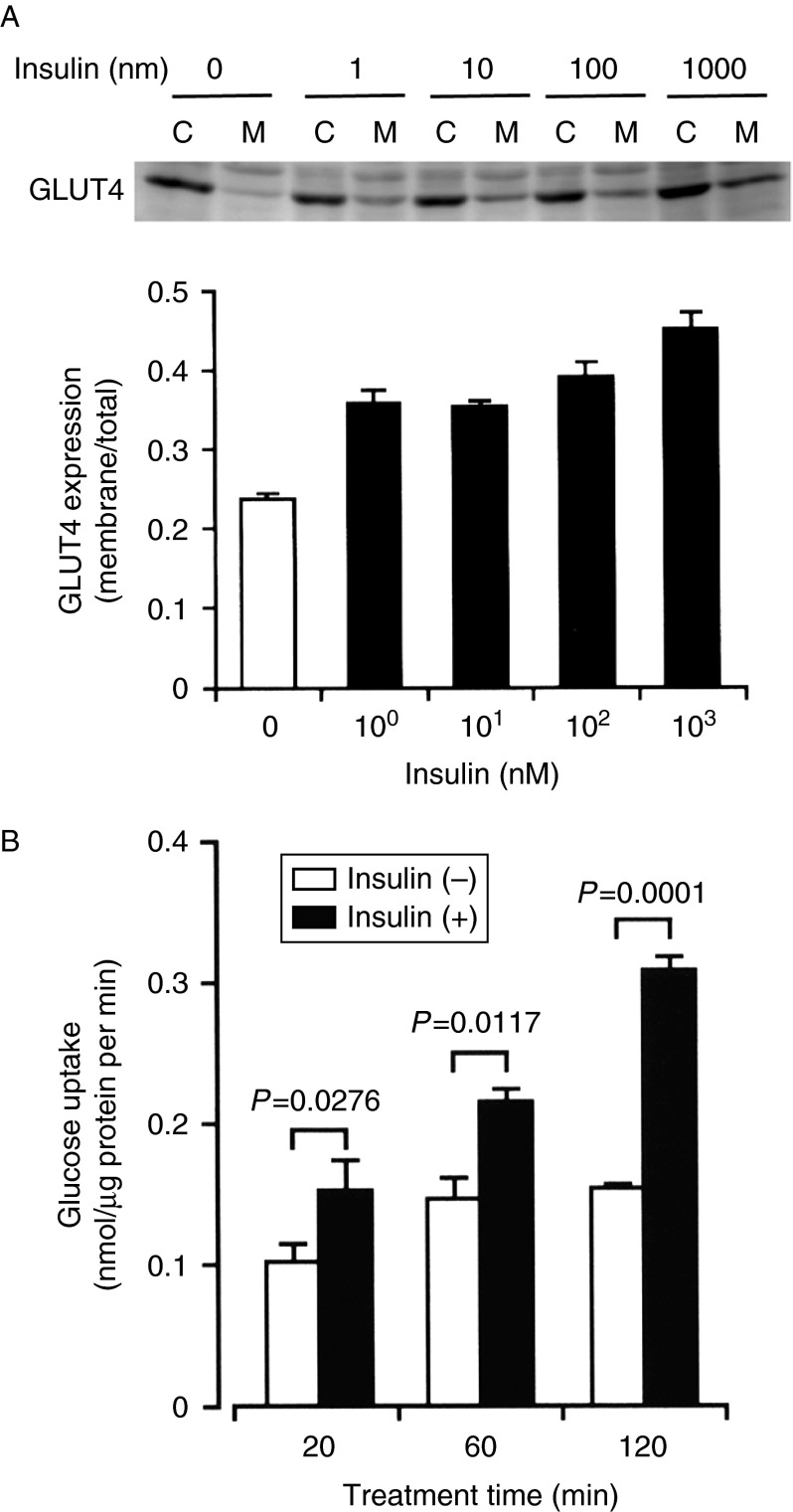
Insulin stimulates GLUT4 translocation toward the cell surface and increases glucose uptake into 3T3-L1-GLUT4myc adipocytes. (A) Cells were left untreated or treated with insulin at the concentrations indicated for 20 min. Then, cells were lysed and separated into the cytosolic (C) and plasma membrane fractions (M), followed by western blotting using antibody against c-myc. In the graph, each column represents the mean (±s.e.m.) ratio of signal intensity for c-myc in the plasma membrane fraction to that in the total cell (*n*=4 independent experiments). (B) Cells were incubated in PBS containing glucose (10 mM) for periods of time as indicated in the presence and absence of insulin (100 nM), and then extracellular glucose was measured by HPLC. In the graph, each column represents the mean (±s.e.m.) glucose uptake (nmol/μg protein per min) (*n*=4 independent experiments). *P* values, unpaired *t*-test.

Insulin promoted GLUT4 translocation from the cytosol to the plasma membrane in 3T3-L1-GLUT4myc adipocytes in a concentration (1 nM–1 μM)-dependent manner (Fig. 2A). In the glucose assay, insulin (100 nM) significantly increased glucose uptake into 3T3-L1-GLUT4myc adipocytes in a treatment time (20–120 min)-dependent manner, the extent reaching approximately twofold more than control levels at 120 min (Fig. 2B). Collectively, these results indicate that insulin stimulates GLUT4 translocation to the cell surface and promotes glucose uptake into adipocytes.

**Figure 3:**
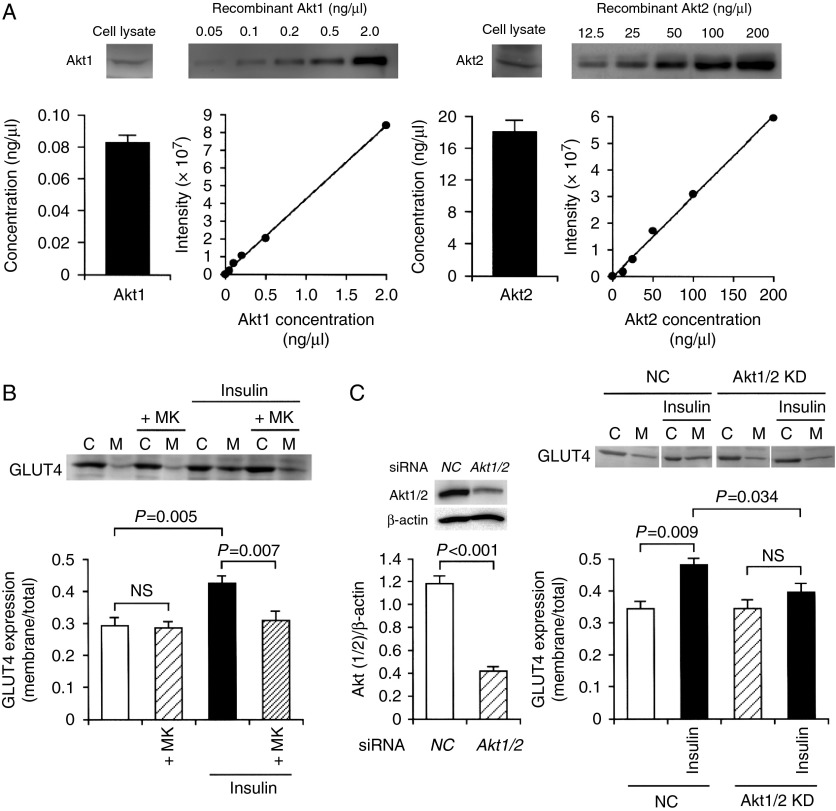
Insulin stimulates GLUT4 translocation toward the plasma membrane in an Akt1/2-dependent manner. (A) The Akt1 or Akt2 concentration/intensity standard curves were made using a human recombinant Akt1 or Akt2 respectively. 3T3-L1-GLUT4myc fibroblasts on day 14 after differentiation induction were lysed followed by western blotting using antibodies against Akt1 and Akt2, and the amount of Akt1 and Akt2 was calculated from each standard curve (*n*=4 independent experiments). (B) 3T3-L1-GLUT4myc adipocytes were treated with insulin (100 nM) in the presence and absence of MK2206 (+MK) (5 μM). Then, cells were lysed and separated into the cytosolic (C) and plasma membrane fractions (M), followed by western blotting using an antibody against c-myc. In the graph, each column represents the mean (±s.e.m.) ratio of signal intensity for c-myc in the plasma membrane fraction to that in the total cell (*n*=4 independent experiments). *P* values, ANOVA followed by a Bonferonni correction. NS, not significant. (C) In the left panel, 3T3-L1-GLUT4myc adipocytes were transfected with the NC siRNA or the Akt1/2 siRNA, and 48 h after transfection western blotting was carried out using antibodies against Akt1/2 or β-actin. Signal intensities for Akt1/2 were normalized to those for β-actin. In the graph, each column represents the mean (±s.e.m.) normalized expression of Akt1/2 (*n*=4 independent experiments). *P* value, unpaired *t*-test. In the right panel, cells transfected with the NC siRNA (NC) or the Akt1/2 siRNA (Akt1/2 KD) were left untreated or treated with insulin (100 nM) for 20 min. Then, cells were lysed and separated into the cytosolic (C) and plasma membrane fractions (M), followed by western blotting using an antibody against c-myc. In the graph, each column represents the mean (±s.e.m.) ratio of signal intensity for c-myc in the plasma membrane fraction to that in the total cell (*n*=4 independent experiments). *P* values, ANOVA followed by a Bonferonni correction. NS, not significant.

Akt plays a central role in insulin-stimulated GLUT4 translocation to the cell surface. The amount of expression of Akt1 and Akt2 in differentiated 3T3-L1-GLUT4myc adipocytes was calculated from the Akt1 and Akt2 concentration/intensity standard curve, respectively. As previously shown (Calera *et al*. 1998), 3T3-L1-GLUT4myc adipocytes expressed both Akt1 and Akt2, but the expression level of Akt2 (18.016±1.487 ng/μl) was much greater than that of Akt1 (0.083±0.005 ng/μl) (Fig. 3A).

The insulin (100 nM)-induced increase in the GLUT4 localization on the cell surface was abolished by the Akt1/2 inhibitor MK2206 (5 μM) (Fig. 3B), indicating that insulin promotes GLUT4 translocation to the cell surface in an Akt1/2-dependent manner. To obtain further evidence for this, we constructed the Akt1/2 siRNA. In the western blot analysis, expression of Akt1/2 protein was apparently reduced in 3T3-L1-GLUT4myc adipocytes transfected with the Akt1/2 siRNA compared with that for cells transfected with the NC siRNA (Fig. 3C), confirming Akt1/2 knock-down. The insulin (100 nM)-induced increase in the GLUT4 localization on the cell surface was significantly suppressed by knocking-down Akt1/2 (Fig. 3C). Taken together, these results indicate that Akt1/2 is a key factor for insulin-stimulated GLUT4 translocation to the cell surface.

### PI3K phosphorylates both Thr308 and Ser473 for Akt1 and Ser474 alone for Akt2

**Figure 4:**
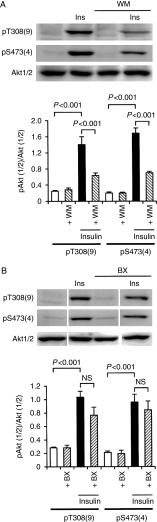
Insulin stimulates phosphorylation of Akt1/2 in a PI3K-dependent manner in 3T3-L1-GLUT4myc adipocytes. Cells were left untreated or treated with insulin (Ins) (100 nM) for 10 min in the presence and absence of wortmannin (+WM) (20 nM) (A) or BX912 (+BX) (100 nM) (B). Western blotting was carried out using antibodies against pT308(9), pS473(4), and Akt1/2. Signal intensities for phosphorylated Akt1/2 (pAkt1/2) were normalized to those for Akt1/2. In the graphs, each column represents the mean (±s.e.m.) normalized intensity for pAkt1/2 at each site (*n*=4 independent experiments). *P* values, ANOVA followed by a Bonferonni correction. NS, not significant.

Insulin (100 nM) increased phosphorylation of Akt1/2 both at Thr308/309 and Ser473/474, indicating that insulin activates Akt1/2 in 3T3-L1-GLUT4myc adipocytes (Fig. 4A and B). Insulin-induced phosphorylation of Akt1/2 at Thr308/309 and Ser473/474 was clearly inhibited by the PI3K inhibitor wortmannin (20 nM) (Fig. 4A). In contrast, the PDK1 inhibitor BX912 (100 nM) also reduced insulin-induced phosphorylation of Akt1/2 at Thr308/309 and Ser473/474, but not significantly (Fig. 4B). These results indicate that in response to insulin PI3K could still activate Akt1/2 in the absence of PDK1 in adipocytes.

**Figure 5:**
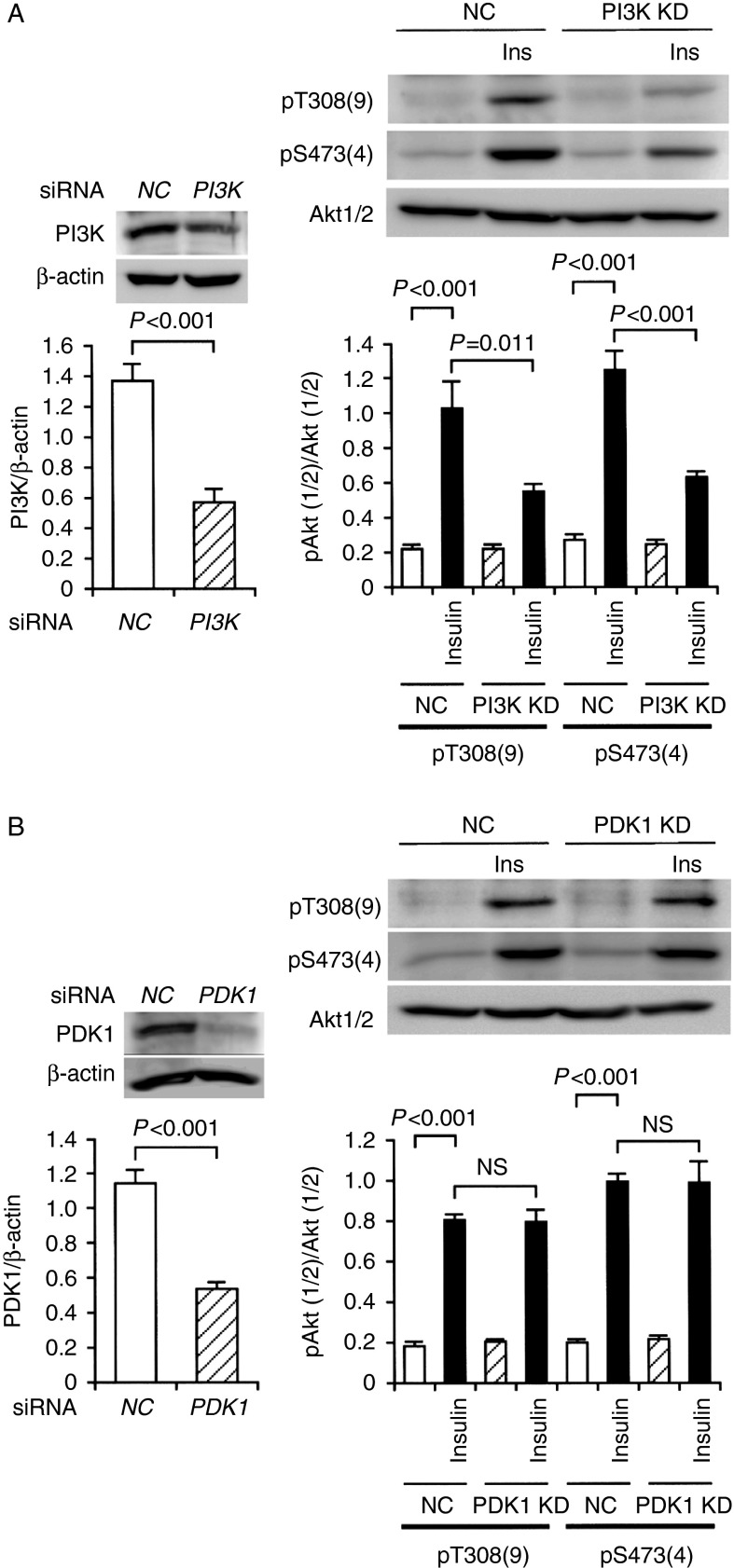
Insulin-induced Akt1/2 phosphorylation at Thr308 and Ser473 was prevented by knocking-down PI3K, but not PDK1, in 3T3-L1-GLUT4myc adipocytes. (A) In the left panel, cells were transfected with the NC siRNA or the PI3K siRNA, and 48 h after transfection western blotting was carried out using antibodies against PI3K or β-actin. Signal intensities for PI3K were normalized to those for β-actin. In the graph, each column represents the mean (±s.e.m.) normalized expression of PI3K (*n*=4 independent experiments). *P* value, unpaired *t*-test. In the right panel, cells transfected with the NC siRNA (NC) or the PI3K siRNA (PI3K KD) were left untreated or treated with insulin (Ins) (100 nM) for 10 min, followed by western blotting using antibodies against pT308(9), pS473(4), and Akt1/2. Signal intensities for phosphorylated Akt1/2 (pAkt1/2) were normalized to those for Akt1/2. In the graph, each column represents the mean (±s.e.m.) normalized intensity for pAkt1/2 at each site (*n*=4 independent experiments). *P* values, ANOVA followed by a Bonferonni correction. (B) In the left panel, cells were transfected with the NC siRNA or the PDK1 siRNA, and 48 h after transfection western blotting was carried out using antibodies against PDK1 or β-actin. Signal intensities for PDK1 were normalized to those for β-actin. In the graph, each column represents the mean (±s.e.m.) normalized expression of PDK1 (*n*=4 independent experiments). *P* value, unpaired *t*-test. In the right panel, cells transfected with the NC siRNA (NC) or the PDK1 siRNA (PDK1 KD) were left untreated or treated with insulin (Ins) (100 nM) for 10 min, followed by western blotting using antibodies against pT308(9), pS473(4), and Akt1/2. Signal intensities for phosphorylated Akt1/2 (pAkt1/2) were normalized to those for Akt1/2. In the graph, each column represents the mean (±s.e.m.) normalized intensity for pAkt1/2 at each site (*n*=4 independent experiments). *P* values, ANOVA followed by a Bonferonni correction. NS, not significant.

To knock-down PI3K and PDK1, we constructed siRNAs for PI3K and PDK1. In the western blot analysis, expression of PI3K and PDK1 proteins was significantly reduced in 3T3-L1-GLUT4myc adipocytes transfected with the PI3K siRNA and the PDK1 siRNA, respectively, as compared with that for cells transfected with each NC siRNA (Fig. 5A and B), confirming knocking-down of PI3K and PDK1. Insulin (100 nM)-induced phosphorylation of Akt1/2 at Thr308/309 and Ser473/474 was apparently suppressed by knocking-down PI3K (Fig. 5A), but otherwise no inhibition was obtained by knocking-down PDK1 (Fig. 5B). This further supports the notion that insulin stimulates Akt1/2 activation in a PI3K-dependent manner, without PDK1, in adipocytes.

**Figure 6:**
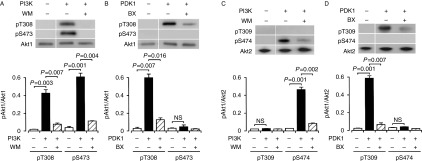
PI3K-dependent and PDK1-independent Akt1 phosphorylation and PI3K-/PDK1-dependent Akt2 phosphorylation under cell-free conditions. Akt1 or Akt2 was reacted with (+) and without (−) PI3K (1 μg/ml) (A and C) or PDK1 (1 μg/ml) (B and D) in the presence and absence of wortmannin (WM) (20 nM) or BX912 (BX) (100 nM), and western blotting was carried out using antibodies against pT308(9), pS473(4), and Akt1/2. Signal intensities for phosphorylated Akt1 (pAkt1) or Akt2 (pAkt2) were normalized to those for Akt1 or Akt2. In the graphs, each value represents the mean (±s.e.m.) intensity for pAkt1 or pAkt2 at each site (*n*=4). *P* values, Dunnett's test. NS, not significant.

In the cell-free assay, PI3K (1 μg/ml) phosphorylated Akt1 both at Thr308 and Ser473, which was abrogated by wortmannin (20 nM) (Fig. 6A). In contrast, PDK1 (1 μg/ml) phosphorylated Akt1 only at Thr308, which was abolished by BX912 (100 nM) (Fig. 6B). Collectively, these results indicate that PI3K is capable of activating Akt1 still in the absence of PDK1. PI3K (1 μg/ml), on the other hand, phosphorylated Akt2 at Ser474 alone, and the effect was abolished by wortmannin (20 nM) (Fig. 6C). PDK1 (1 μg/ml) phosphorylated Akt2 at Thr309 alone, and the effect was completely inhibited by BX912 (100 nM) (Fig. 6D). The activation of Akt2, accordingly, appears to be achieved by PI3K and PDK1.

### PDK1 as well as PI3K also participate in the regulation of GLUT4 translocation to the cell surface

**Figure 7:**
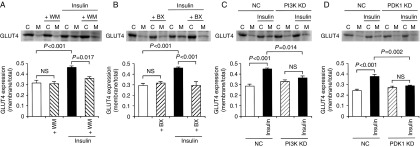
GLUT4 translocation to the cell surface is regulated by PI3K and PDK1. 3T3-L1-GLUT4myc adipocytes were left untreated or treated with insulin (100 nM) in the presence and absence of wortmannin (+WM) (20 nM) (A) or BX912 (+BX) (100 nM) (B) for 20 min. In a different set of experiments, cells transfected with the NC siRNA (NC), the PI3K siRNA (C), or the PDK1 siRNA (D) were treated with insulin (100 nM) for 20 min. Then, cells were lysed and separated into the cytosolic (C) and plasma membrane fractions (M), followed by western blotting using an antibody against c-myc. In the graphs, each column represents the mean (±s.e.m.) ratio of signal intensity for c-myc in the plasma membrane fraction to that in the total cell (*n*=4 independent experiments). *P* values, ANOVA followed by a Bonferonni correction. NS, not significant.

Finally, we examined whether PI3K and PDK1 regulate GLUT4 translocation to the cell surface. Insulin (100 nM)-stimulated GLUT4 translocation to the cell surface in 3T3-L1-GLUT4myc adipocytes was significantly inhibited by wortmannin (20 nM) (Fig. 7A) or knocking-down PI3K (Fig. 7C). Moreover, the insulin (100 nM)-induced increase in the GLUT4 localization on the cell surface was also prevented by BX912 (100 nM) (Fig. 7B) or knocking-down PDK1 (Fig. 7D). Taken together, these results indicate that PI3K and PDK1, a downstream effector of PI3K, are engaged in the insulin-stimulated GLUT4 translocation to the cell surface.

## Discussion

GLUT4 is a member of the facilitative glucose transporter family, characterized by preferential expression in fat and muscle tissues, where it is responsible for insulin-induced glucose uptake into cells. GLUT4 is unique among other members of the GLUT family in its dynamic cycling within adipocytes and muscle cells. Insulin stimulates GLUT4 translocation to the cell surface by activating insulin receptor, and when insulin receptor is inactivated, GLUT4 is rapidly removed from the plasma membrane into the cytosol and accumulated in intracellular organelles such as the trans-Golgi network, recycling endosome, and diverse tubulo-vesicular bodies (Bryant *et al*. 2002, Rudich & Klip 2003, Watson *et al*. 2004, Ishiki & Klip 2005).

**Figure 8:**
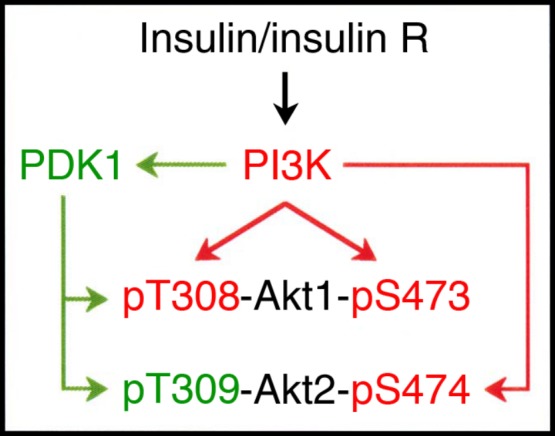
A schematic diagram of Akt activation pathway relevant to insulin signals. Insulin R, insulin receptor.

Akt is a key regulator for insulin-regulated GLUT4 translocation to the cell surface (Cong *et al*. 1997, Wang *et al*. 1999). In this study, insulin promoted GLUT4 translocation from the cytosol to the plasma membrane in a concentration (1 nM–1 μM)-dependent manner and increased glucose uptake into 3T3-L1-GLUT4myc adipocytes. Insulin-induced GLUT4 translocation to the cell surface was clearly inhibited by the Akt1/2 inhibitor MK2206 or knocking-down Akt1/2, indicating that Akt1/2 is required for insulin-stimulated GLUT4 translocation to the cell surface. Accumulating evidence has indicated that Akt is activated via a pathway along an insulin receptor/IRS/PI3K/PDK1/Akt axis (Ishikura *et al*. 2008). Akt1/2 is activated through its phosphorylation at Thr308/309 in the activation-loop of the kinase domain and Ser473/474 in the carboxy-terminal regulatory region (Yang *et al*. 2002, Song *et al*. 2005). In this study, insulin increased phosphorylation of Akt1/2 both at Thr308/309 and Ser473/474 in 3T3-L1-GLUT4myc adipocytes, confirming that insulin activates Akt1/2. Surprisingly, insulin-induced phosphorylation of Akt1/2 was abrogated by the PI3K inhibitor wortmannin or knocking-down PI3K in 3T3-L1-GLUT4myc adipocytes, yet it was not significantly inhibited by the PDK1 inhibitor BX912 or knocking-down PDK1. This suggests that PI3K is indispensable for insulin-induced activation of Akt1/2. In the cell-free assay, PI3K phosphorylated Akt1 both at Thr308 and Ser473, but PDK1 phosphorylated Akt1 only at Thr308. This provides direct evidence that PI3K has the potential to activate Akt1 in a PDK1-independent manner (Fig. 8). PI3K and PDK1, on the other hand, phosphorylated Akt2 at Ser474 and Thr309 respectively. This indicates that Akt2 may be activated by the cooperative mechanism of PI3K and PDK1 (Fig. 8). In this study, Akt2 was present at more than 200-fold greater levels than Akt1 in differentiated 3T3-L1 adipocytes. Then, the critical question is why insulin-induced Akt1/2 phosphorylation at Thr308/309 was not significantly inhibited by BX912 or knocking-down PDK1. A plausible explanation for this is that an anti-pT308(9) antibody might detect Akt1 phosphorylation at Thr308, even though expression levels of Akt1 were quite low. To answer this question, further experiments need to be carried out.

IKBKE and Pak1 have been shown to phosphorylate Akt at Ser473 in a PI3K-independent manner (Mao *et al*. 2008, Guo *et al*. 2011). PI3K, alternatively, activates mTORC2 (Razmara *et al*. 2013), and activated mTORC2 is capable of phosphorylating Akt at Ser473 (Bayascas & Alessi 2005, Gao *et al*. 2005). In this study, PI3K phosphorylated Akt1/2 at Ser473/474 under cell-free conditions in the absence of mTORC2. This indicates that phosphorylation of Akt1/2 at Ser473/474 could be achieved by PI3K alone, although the role of mTORC2 was not investigated. To our knowledge, this is the first study to detect direct phosphorylation of Akt1/2 at Ser473/474 due to PI3K.

The three Akt isoforms Akt1, Akt2, and Akt3 have critical and distinct functions in the regulation of metabolism, cell growth, and apoptosis. A growing body of evidence has argued that Akt2 is required for GLUT4 translocation. Insulin activates both Akt1 and Akt2 in 3T3-L1 and primary rat adipocytes (Bae *et al*. 2003, Gonzalez & McGraw 2009). Isoform-specific activation, therefore, does not account for the selective requirement for Akt2 in the control of GLUT4 translocation to the plasma membrane in adipocytes. Akt2, but not Akt1, is associated with GLUT4 vesicles in biochemical fractionation of adipocytes (Calera *et al*. 1998, Hill *et al*. 1999), suggesting that differential localization allows Akt2 to control isoform-specific signaling. Moreover, insulin induces a preferential accumulation of Akt2, relative to Akt1, at the plasma membrane in adipocytes (Gonzalez & McGraw 2009), indicating that regulation of GLUT4 translocation depends upon the amount of the isoform localization at the plasma membrane, but not the isoform species. In support of this notion, expression of an Akt1 mutant that accumulates at the plasma membrane is sufficient to induce GLUT4 translocation (Gonzalez & McGraw 2009). Akt1, on the other hand, has also been shown to participate in the GLUT4 translocation to the plasma membrane in L6 myoblasts (Wang *et al*. 1999).

In this study, insulin-stimulated GLUT4 translocation to the cell surface in adipocytes was prevented by the Akt inhibitor MK2206 and knocking-down Akt1/2, the PI3K inhibitor wortmannin and knocking-down PI3K, or the PDK1 inhibitor BX912 and knocking-down PDK1. This indicates that insulin stimulates GLUT4 translocation to the cell surface by activating Akt1/2 through a pathway along an IRS/PI3K/PDK1/Akt axis in adipocytes. Akt2 was abundantly expressed in adipocytes and Akt2 activation was achieved by cooperation of PI3K and PDK1 in the cell-free assay. This indicates that insulin dominantly activates Akt2 following activation of PI3K and the downstream effector PDK1 in adipocytes, responsible for GLUT4 translocation. Strangely, insulin-induced phosphorylation of Akt1/2 both at Thr308/309 and Ser473/474 in adipocytes was abrogated by wortmannin and knocking-down PI3K, but otherwise no significant inhibition of Akt1/2 phosphorylation at each site was obtained with BX912 and knocking-down PDK1. A plausible explanation for this is that PDK1 might stimulate GLUT4 translocation to the cell surface by phosphorylating or interacting with an unknown factor, as its downstream target, regardless of Akt1/2 phosphorylation at Thr308/309. To address this, we are currently carrying out further experiments.

In conclusion, the results presented here clearly demonstrate that insulin stimulates GLUT4 translocation to the plasma membrane in a PI3K-, PDK1-, and Akt1/2-dependent manner in 3T3-L1-GLUT4myc adipocytes. The results also show that PI3K phosphorylates Akt1 both at Thr308 and Ser473 and Akt2 at Ser474 and that PDK1, a downstream effector of PI3K, phosphorylates Akt1 and Akt2 at Thr308 and Thr309 respectively. This implies that Akt1 is activated by PI3K alone and that Akt2 activation is achieved by cooperation of PI3K and PDK1. The results of the present study, thus, may extend our understanding about Akt1/2 activation pathways linked to insulin signals.

## Author contribution statement

A Tsuchiya and T Kanno performed all the experiments, analyzed the data, and prepared the figures. T Nishizaki designed the experiments and wrote the manuscript. All authors have approved the final manuscript.
